# Lipidomic analysis of *Arabidopsis* seed genetically engineered to contain DHA

**DOI:** 10.3389/fpls.2014.00419

**Published:** 2014-09-01

**Authors:** Xue-Rong Zhou, Damien L. Callahan, Pushkar Shrestha, Qing Liu, James R. Petrie, Surinder P. Singh

**Affiliations:** ^1^Food Futures National Research Flagship, Commonwealth Scientific and Industrial Research OrganisationCanberra, ACT, Australia; ^2^Plant Industry, Commonwealth Scientific and Industrial Research OrganisationCanberra, ACT, Australia; ^3^Metabolomics Australia, School of Botany, University of MelbourneMelbourne, VIC, Australia; ^4^Centre for Chemistry and Biotechnology, School of Life and Environmental Sciences, Deakin UniversityMelbourne, VIC, Australia

**Keywords:** Lipidomics, metabolic engineering, ω3 LC-PUFA, oilseed, LC-MS, triacylglycerol, *Arabidopsis*

## Abstract

Metabolic engineering of omega-3 long-chain (≥C_20_) polyunsaturated fatty acids (ω3 LC-PUFA) in oilseeds has been one of the key targets in recent years. By expressing a transgenic pathway for enhancing the synthesis of the ω3 LC-PUFA docosahexaenoic acid (DHA) from endogenous α-linolenic acid (ALA), we obtained the production of fish oil-like proportions of DHA in *Arabidopsis* seed oil. Liquid chromatography-mass spectrometry (LC-MS) was used to characterize the triacylglycerol (TAG), diacylglycerol (DAG) and phospholipid (PL) lipid classes in the transgenic and wild type *Arabidopsis* seeds at both developing and mature stages. The analysis identified the appearance of several abundant DHA-containing phosphatidylcholine (PC), DAG and TAG molecular species in mature seeds. The relative abundances of PL, DAG, and TAG species showed a preferred combination of LC-PUFA with ALA in the transgenic seeds, where LC-PUFA were esterified in positions usually occupied by 20:1ω9. Trace amounts of di-DHA PC and tri-DHA TAG were identified and confirmed by high resolution MS/MS. Studying the lipidome in transgenic seeds provided insights into where DHA accumulated and combined with other fatty acids of neutral and phospholipids from the developing and mature seeds.

## Introduction

Metabolic engineering of omega-3 long-chain polyunsaturated fatty acids (ω3 LC-PUFA) in oilseed crops has been attempted by several groups in recent years due to the plateauing in global fishery stocks and the rise in demand for ω3 LC-PUFA containing fish oils (Haslam et al., 2013). Fish oils which contain omega-3 LC-PUFA such as eicosapentaenoic acid (EPA, 20:5ω3) or docosahexaenoic acid (DHA, 22:6ω3) are well known to have anti-inflammatory properties although it is not known if EPA and/or DHA are involved in the suppression of cytokine production (James et al., 2000). The engineered DHA biosynthetic pathway in plant seeds involves multiple steps of desaturation and elongation of fatty acids in phosphatidylcholine (PC) and acyl-CoA pools. We had previously reported the first successful transgenic production of DHA in *Arabidopsis* seed in 2005 (Robert et al., 2005), although only at the level of 0.5% in seed triacylglycerol (TAG). This initial report was followed by the transgenic production of DHA in *Brassica* seed by Wu et al. (2005). Good progress has been made in engineering the ω3 LC-PUFA DHA since then (Petrie et al., 2012; Ruiz-Lopez et al., 2012, 2013b). Recently, we have shown that expressing seven genes for the DHA synthesis pathway in *Arabidopsis* with different seed specific promoters resulted in up to 15% DHA in seed oil (Petrie et al., 2012). This exceeded the 12% level at which DHA is generally found in bulk fish oil.

**Figure 1:**
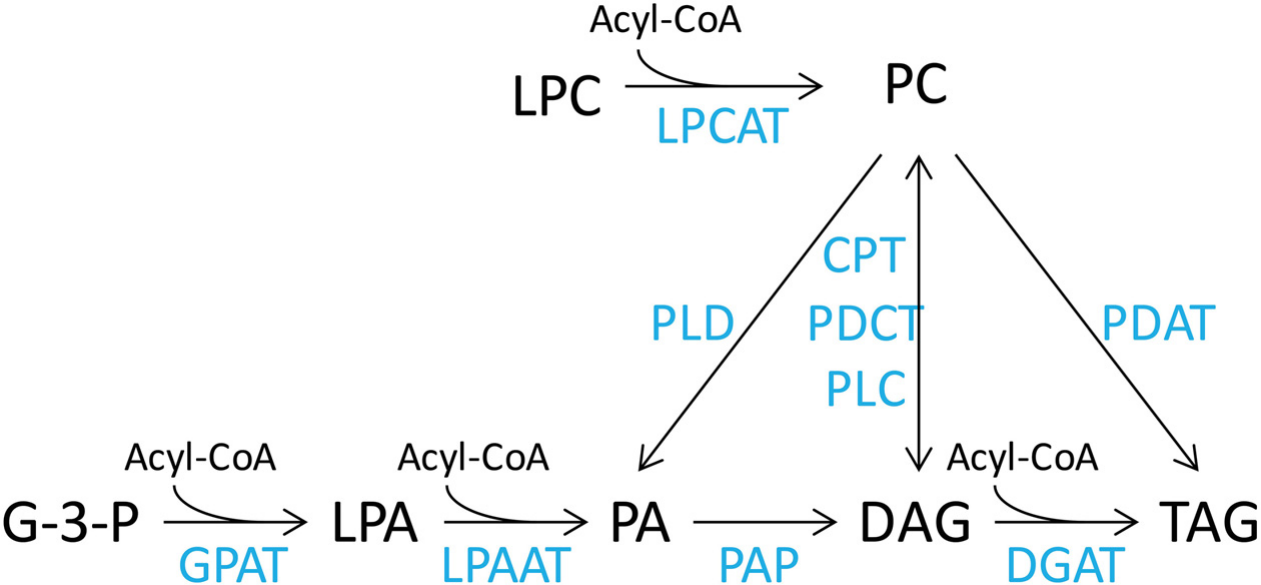
**Simplified representation of lipid metabolism in plant cells**. Lipid pools are acyl-CoA, acyl co-enzyme A; LPC, lysophosphatidylcholine; PC, phosphatidylcholine; G-3-P, glycerol-3-phosphate; LPA, lysophosphatidic acid; PA, phosphatidic acid; DAG, diacylglycerol; TAG, triacylglycerol. The enzymes are LPCAT, lysophosphatidylcholine acyltransferase; PLD, phospholipase D; CPT, CDP-choline:diacylglycerol cholinephosphotransferase; PDCT, phosphatidylcholine:diacylglycerol cholinephosphotransferase; PLC, phospholipase C; PDAT, phospholipid:diacylglycerol acyltransferase; GPAT, glycerol-3-phosphate acyltransferase; LPAAT, lysophosphatidic acid acyltransferase; PAP, phosphatidic acid phosphatase; DGAT, diacylglycerol acyltransferase.

The DHA synthesis pathway consists of a series of fatty acid desaturation and elongation steps that are thought to occur in discrete seed lipid pools. Therefore, effects of transgenic expression of this pathway are expected to be expressed throughout the lipidome of the developing seed. Oil accumulation in the developing seed requires cross talk between the ER membrane lipids, predominantly represented by PC and other phospholipids, such as phosphatidic acid (PA), phosphatidylethanolamine (PE), phosphatidylglycerol (PG), phosphatidylinositol (PI) and phosphatidylserine (PS), soluble acyl CoA fatty acids and DAG lipid pools. The fatty acids in the acyl-CoA pool are then utilized by the acyl-CoA-dependent Kennedy pathway to be assembled into TAG. This involves the sequential acylation of glycerol-3-phosphate to produce phosphatidic acid (PA) by glycerol-3-phosphate acyltransferase (GPAT) and 1-acyl-glycerol-3-phosphate acyltransferase (LPAAT), dephosphorylation of PA to diacylglycerol (DAG) by phosphatidic acid phosphatase (PAP), and the final acylation of DAG to TAG by diacylglycerol acyltransferase (DGAT) (Figure 1). In addition, *de novo* synthesized DAG can be used for synthesis of PC by a reversible cytidine-5′-diphosphocholine:diacylglycerol cholinephosphotransferase (CPT) (Slack et al., 1983). However, recent studies (Williams et al., 2000; Bates et al., 2012; Wang et al., 2012) have demonstrated that the acyl-editing cycle is the major pathway for PC synthesis. This cycle proceeds through the acylation of lysophosphatidylcholine (LPC) and deacylation of PC by acyl-CoA:lysophosphatidylcholine acyltransferase (LPCAT) (Stymne and Stobart, 1984; Bates et al., 2012; Wang et al., 2012). On the other hand, there is accumulating evidence of the use of PC-derived DAG for synthesis of PUFA-containing TAG in plants (Bates and Browse, 2012). The PC to DAG conversion is believed to be mainly carried out by phosphatidylcholine:diacylglycerol cholinephosphotransferase (PDCT) through phosphocholine head group exchange (Bates and Browse, 2012; Hu et al., 2012), although PC-derived DAG can also be synthesized by CPT, or phospholipase C.

As it is proposed that the fatty acyl chains in the PC and DAG pools direct the flux of engineered LC-PUFA products onto TAG, a detailed analysis of the lipids in TAG, DAG and PL in control and transgenic *Arabidopsis* seeds during seed development was required. Traditional gas chromatography (GC) analysis or thin layer chromatography (TLC) coupled with GC can provide an overall profile for each separate class. In the transgenic line GA7 expressing the entire DHA pathway, it was not clear how newly synthesized LC-PUFA's are directed toward the synthesis of neutral lipids, in particular their assembly in TAGs. Recently developed lipidomics techniques using liquid chromatography-mass spectrometry (LC-MS) now provides a detailed composition of the majority of individual lipid molecular species and lipid classes present in cells and tissues (Han and Gross, 2005; Blanksby and Mitchell, 2010). This information will help elucidate the metabolic pathways and potential bottlenecks where the engineered fatty acids accumulate and combined with other endogenous fatty acids. The lipidomics analysis techniques have been successfully used in many applications (Watson, 2006; Ferreri and Chatgilialoglu, 2012; Maatta et al., 2012; Hyötyläinen et al., 2013). In this study we utilized the different scan functions of a triple quadruple-MS (QQQ-MS), such as neutral loss, or precursor ion scan and targeted multiple reaction monitoring (MRM) to characterize the lipid profiles. A quadruple time of flight-MS (QTOF-MS) was also used to confirm the identities of some important lipid species.

## Materials and methods

### Plant materials

*Arabidopsis thaliana* Columbia (Col-0) wild type (WT) and DHA producing transgenic line GA7 (Petrie et al., 2012) were grown in a glasshouse at 22°C and a 16/8 h light and dark cycle. GA7 was generated by expressing the *Lachancea kluyveri* Δ 12-desaturase, *Pichia pastoris* Δ 15-/ω3-desaturase, *Micromonas pusilla* Δ 6-desaturase, *Pyramimonas cordata* Δ 6- and Δ 5-elongases and *Pavlova salina* Δ 5- and Δ 4-desaturases. It has been shown previously that expression of these genes results in the accumulation of up to 15.1% DHA in total seed oil (Petrie et al., 2012). Developing seeds were harvested on dry ice at 10–12 days after flowering (DAF), frozen with liquid nitrogen and freeze dried prior to lipid analysis.

### Total lipid extraction

Total lipids were extracted using methanol/chloroform from triplicate samples of the developing and the mature seeds (Zhou et al., 2013). Tri-17:0-TAG (Nuchek Prep, Elysian, MN, USA) was added based on the dry weight of developing or mature seeds, as an internal standard. Aliquots of the total lipid extracts from 1 mg of seeds (dry weight) were dried under N_2_ followed by re-dissolving in 1 mL of butanol:methanol (1:1, v/v) containing 10 mM butylated hydroxytoluene prior to LC-MS analysis. The concentration of the lipids were measured and the relative amounts based on the same dry weight of seed were compared.

### Two-dimensional TLC

The two-dimensional TLC analysis was carried out in one of the triplicate samples. Individual chloroplastidic and extra-chloroplastidic polar lipid classes were separated from the total lipids using two-dimensional TLC using chloroform:methanol:water (65:25:4, v/v/v) as the first solvent system and chloroform:methanol:NH_4_OH:ethylpropylamine (65: 35:5:0.5, v/v/v/v) as the second solvent system as described (Khozin et al., 1997). The lipid spots were visualized under UV after spraying 0.001% primuline in acetone/water (4:1, v/v) and were collected for GC analysis with a known amount of 17:0 free fatty acid as internal standard as described previously (Zhou et al., 2013).

### Lipid profiling with LC-MS

The extracted total lipids were analyzed using an Agilent 1200 series LC coupled to an Agilent 6410B electrospray ionization QQQ-MS (Agilent, Palo Alto, California, USA). A 5 μL injection of each total lipid extract was chromatographically separated with an Ascentis Express RP-Amide 50 × 2.1 mm, 2.7 μm HPLC column (Sigma-Aldrich, Castle Hill, Australia) using a binary gradient with a flow rate of 0.2 mL/min. The mobile phases were: A. 10 mM ammonium formate in H_2_O:methanol:tetrahydrofuran (50:20:30, v/v/v.); B. 10 mM ammonium formate in H_2_O:methanol:tetrahydrofuran (5:20:75, v/v/v.). Selected neutral lipids (TAG and DAG) and phospholipids (PL, including PC, PE, PI, PS, PA, PG) were analyzed by multiple reaction monitoring (MRM) using a collision energy of 30 V and fragmentation energy of 60 V. Neutral lipids were targeted on the following major fatty acids: 16:0 (palmitic acid), 18:0 (stearic acid), 18:1ω9 (oleic acid, OA), 18:2ω6 (linoleic acid, LA), 18:3ω3 (α-linolenic acid, ALA), 18:4ω3 (stearidonic acid, SDA), 20:1, 20:2, 20:3, 20:4ω3, 20:5ω3, 22:4ω3, 22:5ω3, 22:6ω3, while phospholipids were scanned containing C_16_, C_18_, C_20_, and C_22_ species with double bonds of 0–3, 0–4, 0–5, 4–6 respectively. Individual MRMs for each TAG was based on ammoniated precursor ion and product ion from neutral loss of 20:1, SDA, EPA, and DHA. TAG and DAG were quantified using the 50 μ M tristearin and distearin injected in the same batch as external standards. Phospholipids were quantified with 10 μM of di-18:0-PC, di-17:0-PA, di-17:0-PE, 17:0–17:1-PG, di-18:1-PI and di-17:0-PS external standards (Avanti Polar Lipids, Alabaster, Alabama, USA) injected in the same batch, and presented as μM in the total lipid extract. All the LC-MS data were presented as average of triplicate samples with standard deviation, calculated with Microsoft Excel. Selected TAG, DAG, and PL species were further confirmed by Agilent 6520 Q-TOF MS/MS using the same chromatographic conditions as just described.

## Results and discussion

### Membrane phospholipid characterization: increased phospholipids in transgenic line

We previously showed that expressing seven enzymes for DHA biosynthesis pathway in *Arabidopsis* resulted in the efficient accumulation of DHA (up to 15.1%) in total seed lipids of transgenic line GA7 (Petrie et al., 2012). These enzymes included *L. kluyveri* Δ 12-desaturase, *P. pastoris* Δ 15-/ω3-desaturase, *M. pusilla* Δ 6-desaturase, *P. cordata* Δ 6- and Δ 5-elongases and *P. salina* Δ 5- and Δ 4-desaturases. GA7 showed significantly increased ALA and a reduced proportion of 20:1 in total seed lipids. In addition, GA7 accumulated only very low amounts of C_20_ intermediates. In the present study, we have further characterized the PL, DAG, and TAG species in detail using LC-MS analysis in MRM mode. Comparative LC-MS analysis was carried out on the developing and mature seeds of GA7 and wild type (WT) control in order to compare the lipid species.

An overall increase in PL was observed in GA7 when compared to the wild type seeds from the plants grown at the same time. The profiles and the amounts of the different PL classes were first analyzed by two-dimensional TLC followed by fatty acid analysis by GC, as the indicative result. The most abundant PL species, PC, in the *Arabidopsis* seeds, significantly increased (40%) in GA7 mature seeds compared to WT mature seeds as shown in Table 1, based on seed dry weight. Detailed lipidomic profiling was then carried out using LC-MS on triplicate samples. The LC-MS data showed even higher than 40% increase of PC from WT to GA7 (see below). It has been shown in multiple plants that fatty acid engineering can reduce total TAG production and thus reduce seed weight (Dauk et al., 2007; Li et al., 2012; Bates et al., 2014). The expression of DHA synthesis pathway in Columbia did reduce the mature seed weight from 2.05 ± 0.13 mg in WT to 1.89 ± 0.03 mg in GA7 per 100 seeds (five replicates). In other words, GA7 resulted in about 8% reduction in seed weight compared to WT. Although we did not analyse the lipids concentration per individual seed, it is clear that the 8% reduction of seed weight compared with the 40% increase in PC from WT to GA7 still shows an increase in the levels of PC even on a per seed base. Both the WT and GA7 showed higher amounts of PC in the developing seeds (WT-d, GA7-d) than in their mature seeds (WT-m and GA7-m). Further, GA7 had increased total amounts of PC in the developing (2.3-fold higher) and mature seeds (1.4-fold higher) than the WT mature seeds. Again, the amount of PC was expressed based on seed dry weight. Nevertheless, ectopic expression of five membrane-associated fatty acid desaturases resulted in an altered unsaturated fatty acid profile of the PC fraction in GA7. The levels of oleic acid and linoleic acid in PC showed a significant reduction, with decreased of 10.5–0.4 and 47.4–1.4%, respectively. This is associated with a significant increase of newly synthesized ω3 and ω6 LC-PUFA. This result was confirmed by more detailed LC-MS analysis of PC species. Other PL classes followed a similar pattern of increase in GA7 compared to WT as described for PC, and altered the fatty acid profile similar to PC, i.e., a significant reduction of 18:2 and an associated increase of 18:3 and LC-PUFA based on seed dry weight (Table S1, and see detail LC-MS analysis below).

**Table 1:** **Fatty acid profile of phosphatidylcholine in *Arabidopsis* seeds**.

**Sample**	**16:0**	**16:1**	**16:3**	**18:0**	**18:1**	**18:1**	**18:2**	**20:0**	**20:1**	**20:1**	**22:0**	**22:1**	**20:2**	**24:0**	**24:1**	**18:3**	**18:3**	**18:4**	**20:3**	**20:4**	**20:5**	**22:3**	**22:5**	**22:6**	FA^*^
					**ω9**	**ω7**			**ω9**	**ω7**			**ω6**			**ω6**	**ω3**	**ω3**	**ω3**	**ω3**	**ω3**	**ω3**	**ω3**	**ω3**	**(mg)**
WT-d	20.4	0.4	0.1	2.6	3.1	1.5	50.8	0.3	1.3	0.1	0.4	0.1	1.6	1.0	0.2	0.0	15.4	0.0	0.6	0.0	0.0	0.0	0.0	0.0	0.54
WT-m	10.3	1.6	1.4	2.6	10.5	2.5	47.7	0.2	4.4	0.6	0.1	0.2	1.5	0.3	0.2	0.0	15.5	0.0	0.2	0.0	0.0	0.1	0.0	0.0	0.46
GA7-d	16.5	0.3	0.2	2.6	3.7	3.0	21.3	0.2	2.0	0.2	0.2	0.1	0.9	0.4	0.1	0.3	40.6	1.6	1.2	0.3	0.7	0.0	2.1	1.4	1.03
GA7-m	14.8	0.2	1.1	3.8	0.4	4.6	1.4	0.4	1.2	0.7	0.1	0.1	0.8	0.3	0.2	0.3	38.2	8.2	2.8	1.1	1.0	0.0	6.2	11.7	0.64

* mg FA/100 mg dry seed.

**Figure 2:**
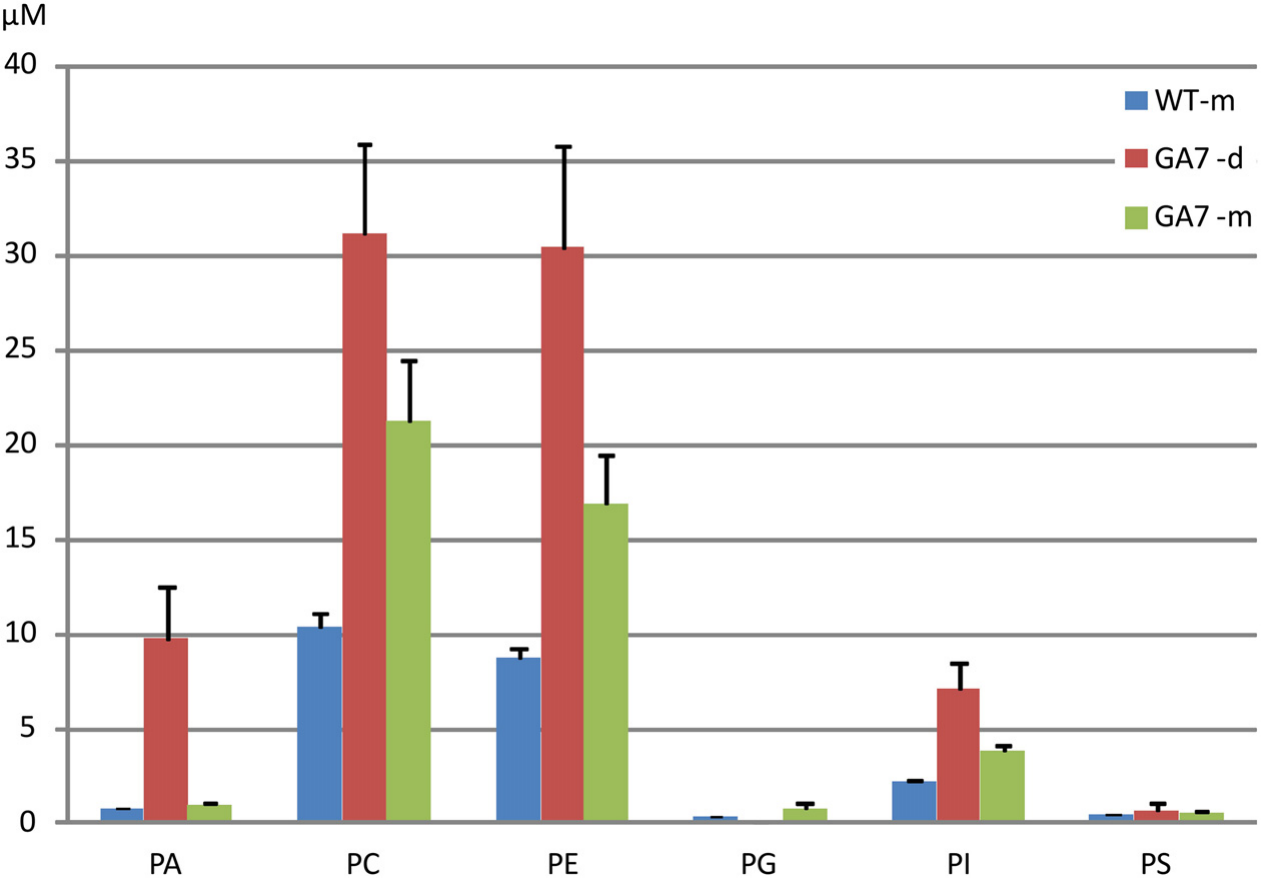
**Total phospholipids in developing and mature *Arabidopsis* seeds**. Phosphatidic acid (PA), phosphatidylcholine (PC), phosphatidylethanolamine (PE), phosphatidylglycerol (PG), phosphatidylinositol (PI) and phosphatidylserine (PS) were determined from triplicate samples with the mean and standard deviation shown. WT-m, Columbia wild type mature seeds; GA7-d and GA7-m, developing and mature seeds of transgenic GA7 line containing a DHA biosynthetic pathway (genes are described in the text). Data are shown as the mean of triplicate analysis with the error bars representing the standard deviations, concentrations (μM) have been determined in the extracts from the same amount of dry seed weight for both developing and mature seeds.

LC-MS analysis of the PL was focused on PA, PC, PE, PG, PI, and PS. The major fatty acids from palmitic acid to the end product ω3 DHA in the engineering pathway were analyzed. As shown in Figure 2, PC and PE were the major PL, followed by PA, with very low amounts of PG and PS. The LC-MS analysis confirmed that there were more total PLs in the developing seeds than in the mature seeds of GA7.

**Figure 3:**
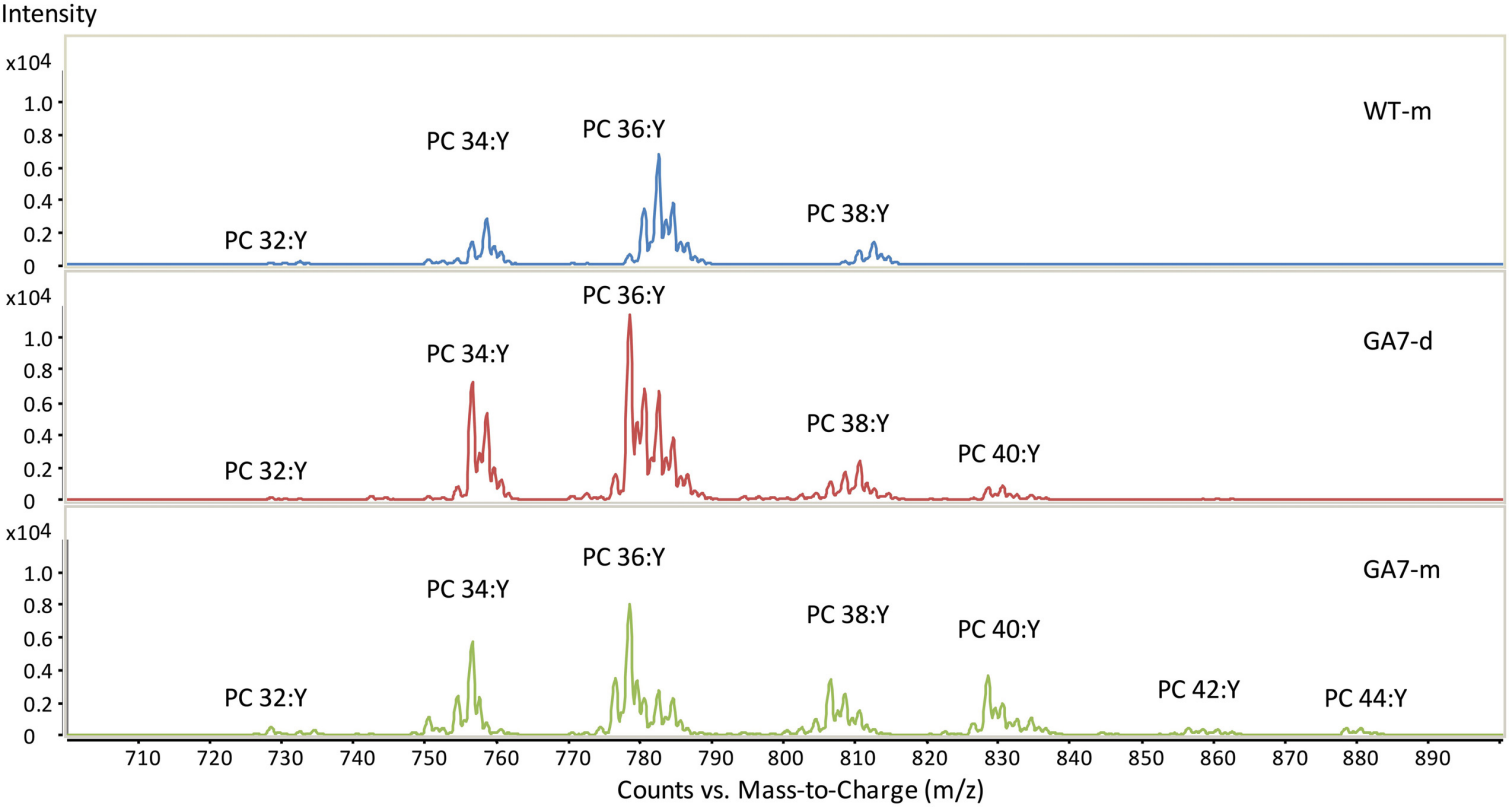
**Summed spectra from the precursor ion scan for phosphatidylcholine (PC) from the WT or GA7 *Arabidopsis* seeds**. PC 36:Y indicates PC species with a total of 36 carbons with a total number of Y double bonds in the two acyl chains. Y axis represents the response of ion scan.

**Figure 4:**
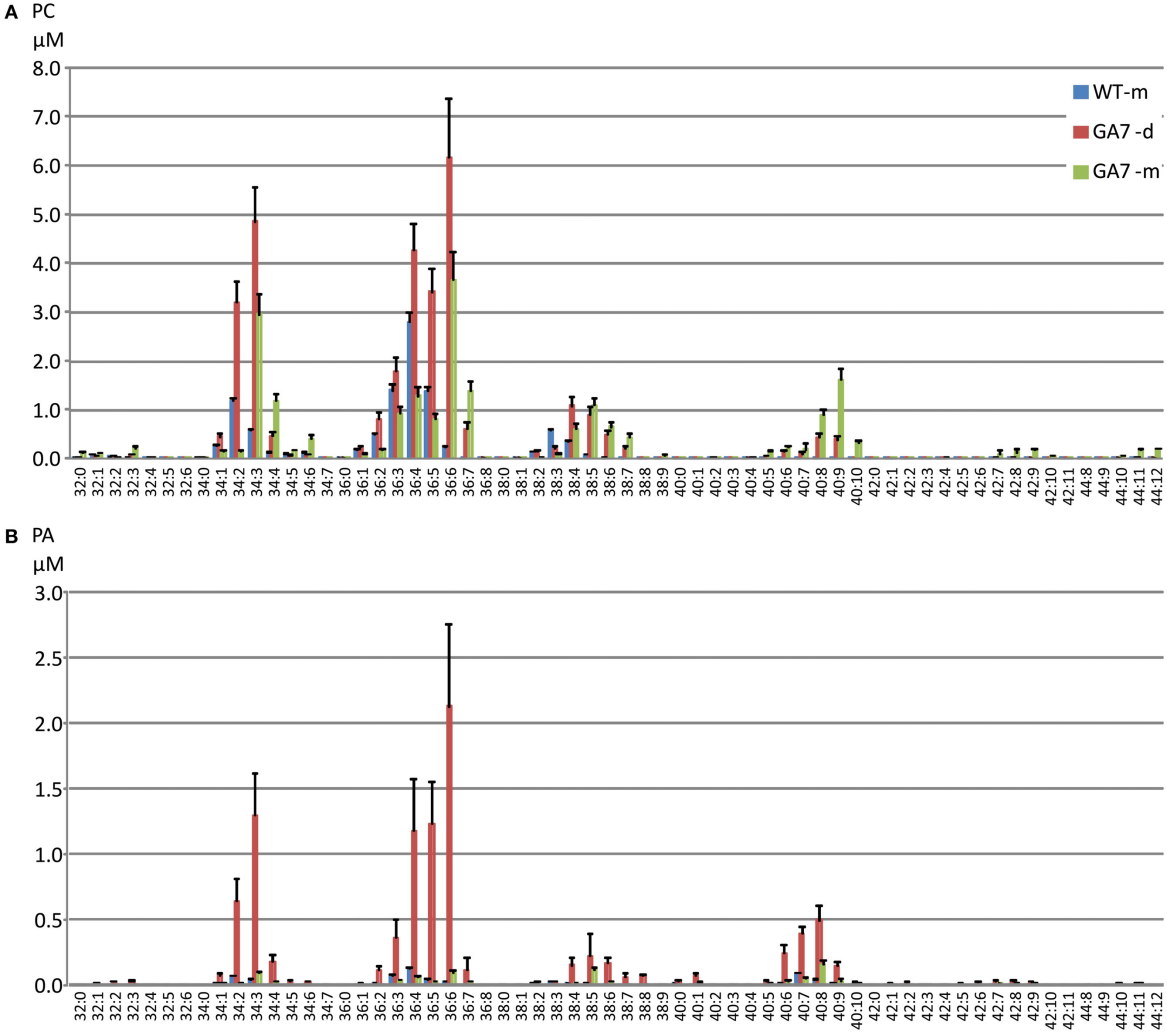
**Distribution of phosphatidylcholine (PC) and phosphatidic acid (PA) species in the developing and mature seeds from the WT or GA7 *Arabidopsis* seeds**. The PC species **(A)** and PA species **(B)** are annotated as X:Y where X is the total carbon number and Y is the total double bond number in the two acyl chains. Data are shown as the mean of triplicate analysis with the error bars representing the standard deviations.

Both the developing and mature seeds of GA7 produced new LC-PUFA in phospholipids. PC precursor total ion scan showed that the WT seeds contained PC with total acyl chain lengths only up to C_38_ (PC 38:Y, in which a total of 38 carbons occurs in the two acyl chains on PC with a total double bond number of Y). The most likely fatty acid combination of being C_18_ and C_20_ (Figure 3). The WT only had low amounts of C_20_ fatty acids in PC (Table 1). The developing GA7 seeds produced PC 40:Y, indicating the esterification of two C_20_ fatty acids. In the mature GA7 seeds, there were low yet detectable amounts of PC 42:Y and PC 44:Y, i.e., PC C_20_/C_22_ or PC C_22_/C_22_, indicating the accumulation of LC-PUFA in the PC pool. Furthermore, precursor ion scan showed higher abundance of the earlier eluted part of the each PC chain length group in GA7 when compared to WT. The molecular species with more double bonds eluted slightly earlier than the molecular species with fewer double bonds in the group with same chain length. This indicated that the PC clusters in GA7 contain more double bonds. This was further supported by the quantification of the different PC species as shown in Figure 4A.

There was a large increase in PC 36:6 (PC 18:3/18:3 as the major species) in GA7 seeds, followed by PC 34:3 (PC 16:0/18:3 as the major species). PC species containing higher numbers of carbons and double bonds in their acyl chains such as PC 40:4 to PC 44:12 were only found in GA7, suggesting EPA or DHA also accumulated in the PC pool. There was a small amount of PC 44:12 (0.9 ± 0.01% out of total PC in mature GA7 seeds), which was likely PC 22:6/22:6. MS/MS analysis of this precursor (878.6 *m/z*) by Q-TOF confirmed the loss of PC head group (184.2 *m/z*) and the 22:6 fatty acid (568.3 *m/z*), as shown in Figure S1.

There was a much lower level of PA in the mature seeds than in the developing seeds based on dry seed weight (Figure 2). It should be noted that lipase activity during extraction could contribute to the higher amount of PA in developing seeds, through conversion of PC to PA, and therefore a reduction of the PC concentration would be expected. Two extraction methods were tested using either hot isopropanol quenching (either at 85 or 95°C) or with the procedure described above, and no significant difference in PA concentrations was found by these two methods. Our results showed both PA and PC (and even PE, PI) were higher in developing seeds than in mature seeds (Figure 2), suggesting the higher amount of PA was not an artifact from lipase activity. GA7 had significantly higher amounts of some PA species, especially PA 36:6, PA 36:5, PA 36:4, PA 34:3 and PA 40:8 than other species (Figure 4B). Similar to PC, PA species containing higher numbers of carbons and double bonds in their acyl chains, such as PA 40:6 to PA 40:9, were only found in GA7.

**Figure 5:**
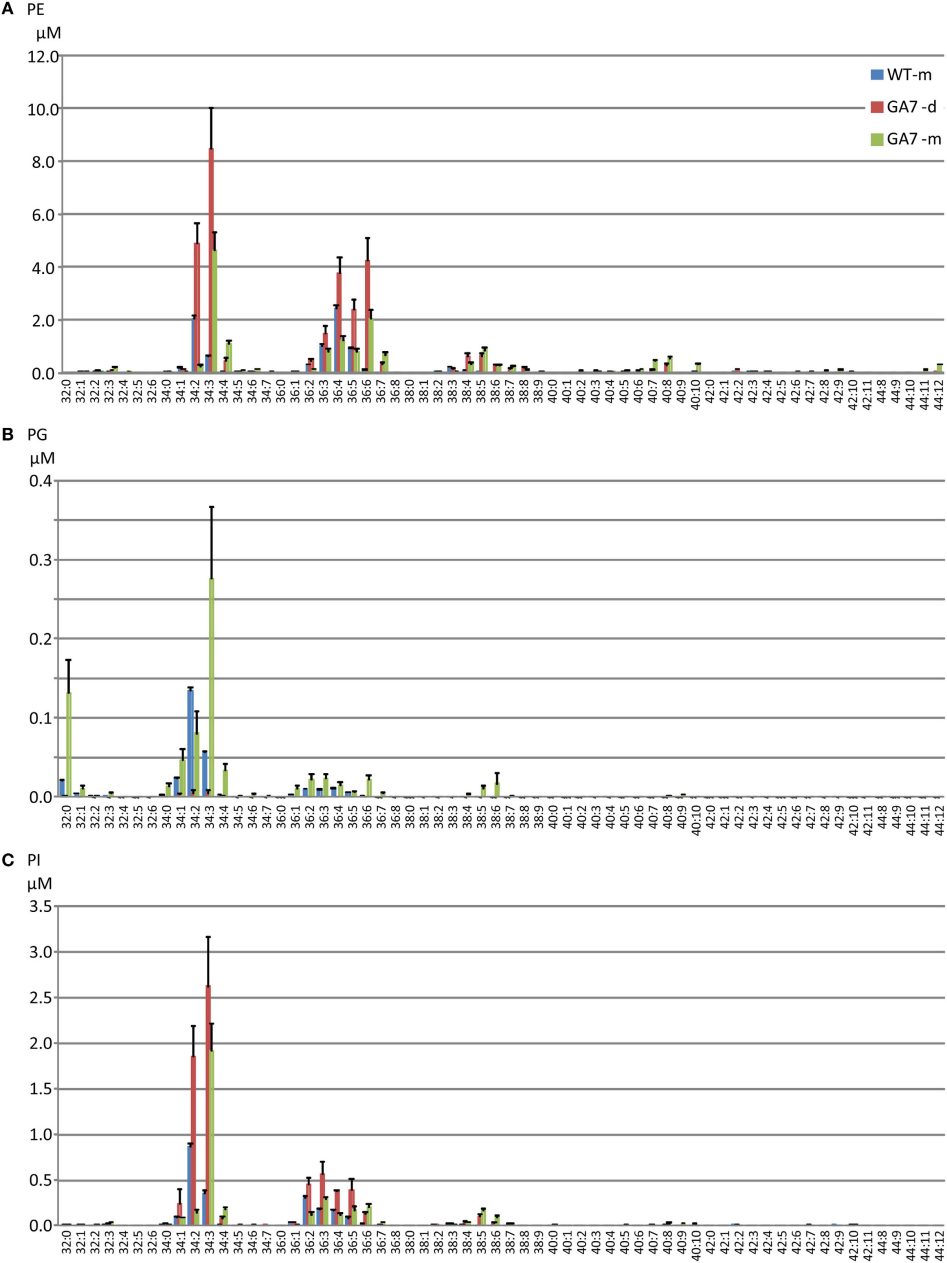
**Phosphatidylethanolamine (PE), phosphatidylinositol (PI) and phosphatidylglycerol (PG) pools in the developing and the mature *Arabidopsis* seeds**. The phospholipids PE **(A)**, PG **(B)** and PI **(C)** species are annotated similarly to PC. Data are shown as the mean of triplicate analysis with the error bars representing the standard deviations.

LC-PUFA in other PL groups were also analyzed (Figure 5). PE was the second largest pool of PL. However, only low levels of LC-PUFA appeared in the PE pool. LC-PUFA was much lower in the PI and PG pools. Interestingly, the most significant increase in these three pools of PL in GA7 was 34:3 (possibly composed of palmitic acid and ALA) when compared to the WT. Another interesting aspect was that the much lower levels of PG was found in the GA7 developing seeds, compared to PE and PI. The PS pool was very small, and the LC-MS responses to most of the PS species were close to the detection limit (see Figure S2).

The substantially increased concentration of membrane PL in GA7 seeds when compared to WT seeds, especially the higher levels of PL in developing seeds compared to mature seeds, could be due to the over-expression of the introduced membrane bound fatty acid desaturases and elongases. In higher plants, acyl groups esterified onto PC are the substrates of desaturation by ER membrane desaturases (Sperling et al., 1993). Acyl editing via a PC-deacylation and lysophosphatidylcholine (LPC)-reacylation cycle provided LC-PUFA in the acyl-CoA pool, for further desaturation and elongation by the introduced acyl-CoA dependent enzymes, and for TAG assembly via the Kennedy pathway. The PC-deacylation and LPC-reacylation cycle was catalyzed by acyl-CoA:LPC acyltransferase (LPCAT) (Bates et al., 2012). Although the engineered pathway mainly targeted acyl-CoA desaturases and elongases, the substantially increased PC pool suggested the importance of the role of PC in accumulation of LC-PUFA in TAG. This suggestion is also supported by Bates and colleagues who showed that the DAG used for TAG synthesis was mostly derived from PC (Bates et al., 2009).

### Accumulation of LC-PUFA containing DAG and TAG in transgenic seeds

Plants commonly have neutral lipid pools in seeds consisting of C_16_ to C_22_ fatty acids with zero to three double bonds. *Arabidopsis* seeds have low amounts of unsaturated C_16_ fatty acids and C_22_ fatty acids. However, due to the expression of the DHA biosynthesis pathway, there can be more than 20 individual fatty acids in the seed lipids. The total number of theoretical TAG species is 8000 (20 × 20 × 20). We therefore targeted only the neutral lipid DAG and TAG species that contain at least one acyl chain consisting of 20:1, SDA, EPA, or DHA by setting up MRM's with neutral losses containing one of the four fatty acids of interest. These are shown in the format of TAG 20:1/X:Y, or TAG DHA/X:Y, where X and Y are the total numbers of carbons and double bonds of fatty acids on the remaining two positions (one position for DAG), respectively.

**Figure 6:**
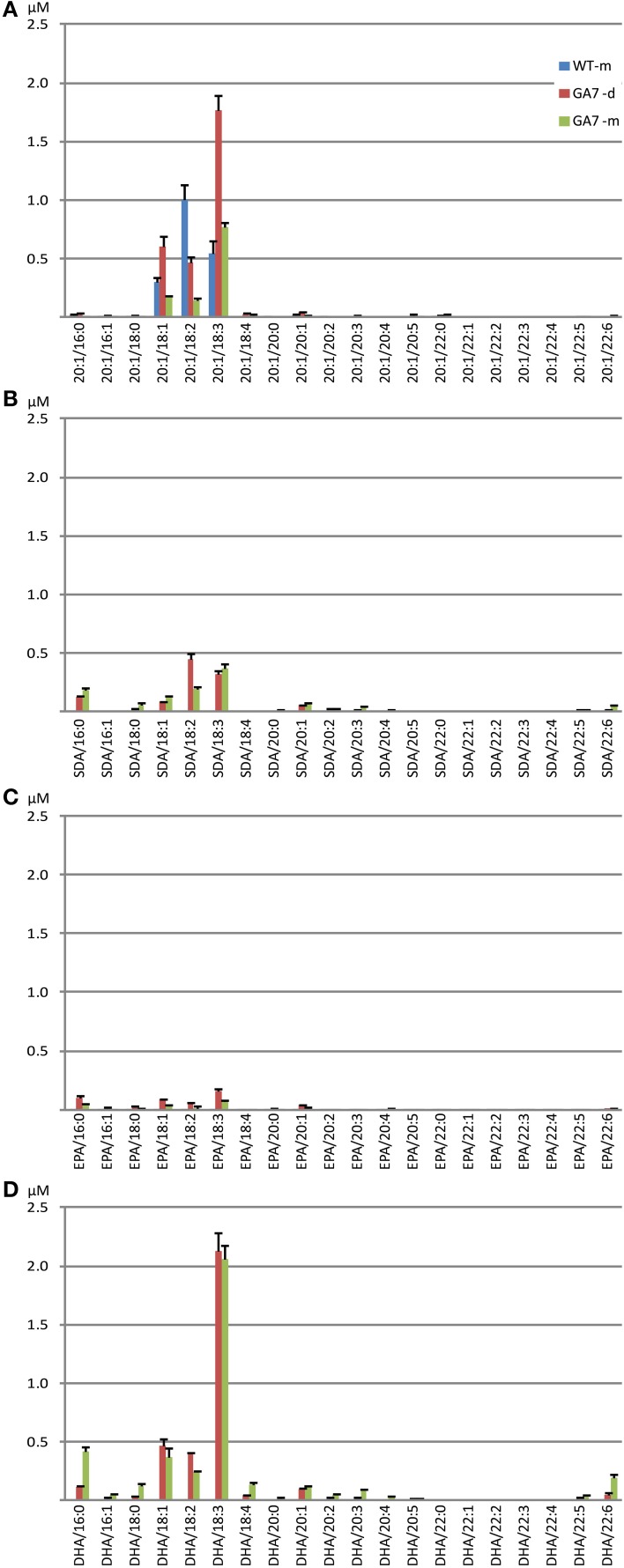
**Abundance of different diacylglycerol (DAG) species in the WT and the GA7 *Arabidopsis* seeds**. DAG species are labeled in format of DAG target/X:Y, where target will be 20:1 **(A)**, SDA **(B)**, EPA **(C)** or DHA **(D)**, with another acyl containing X number of carbons and Y number of double bonds. Data are shown as the mean of triplicate analysis with the error bars representing the standard deviations.

Analysis of DAG species containing one of the four targeted fatty acids showed that both the GA7 developing and mature seeds accumulated substantial amounts of DHA-containing DAG, with low amounts of DAG containing the intermediate SDA or EPA (Figure S3). In WT, the most abundant 20:1-containing DAG was DAG 20:1/18:2 (52.2% among all 20:1-containing DAG), followed by DAG 20:1/18:3. In contrast, the GA7 mature seeds showed about five times more DAG 20:1/18:3 than DAG 20:1/18:2 (65.0% vs. 12.2% among all 20:1-containing DAG). The major DAG species among all 4 analyzed DAG groups were the DAG molecules containing C_18:3_(Figure 6). In GA7 mature seed lipids, a relatively high percentage of DAG SDA/16:0 and DAG EPA/16:0 was also found, making up 16.0% of total SDA-containing DAG and 23.0% of total EPA-containing DAG, although the overall amounts were low (Figures 6B,C). In contrast, DAG DHA/16:0 was found only at 10.7% of the total DHA-containing DAG, while the predominant DAG DHA/18:3 species was 52.0%.

**Figure 7:**
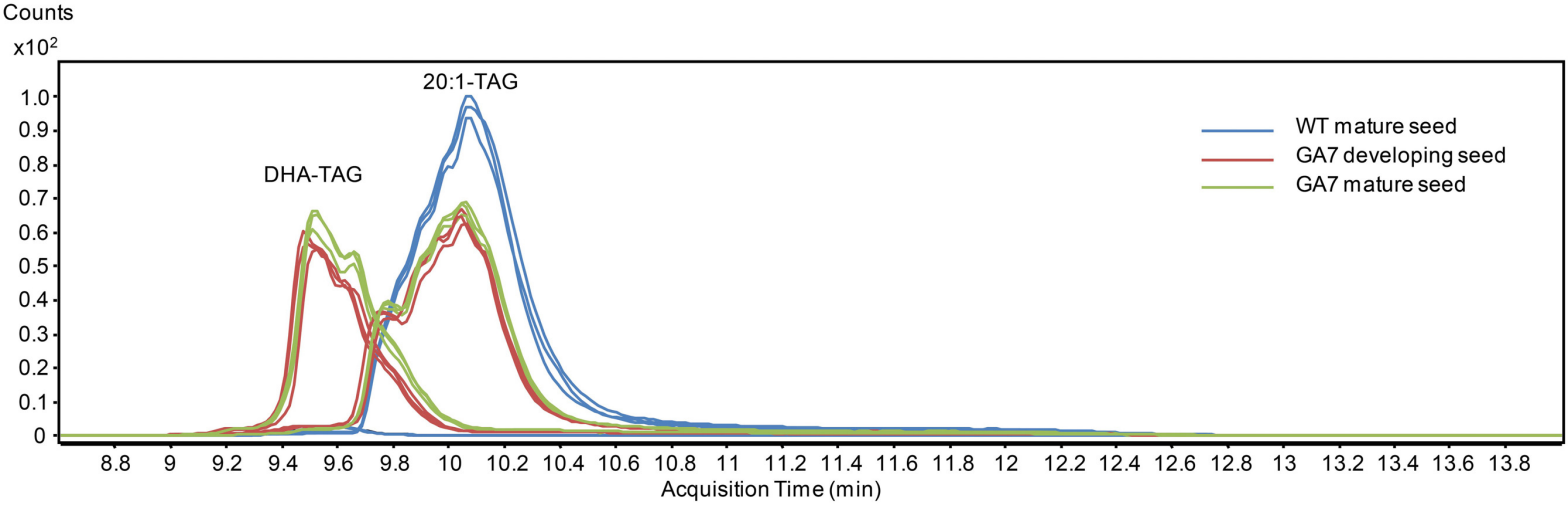
**Total ion chromatogram from multiple reaction monitoring (MRM) of the triacylglycerol (TAG) species containing 20:1 or DHA from the WT and the GA7 *Arabidopsis* seeds**.

**Figure 8:**
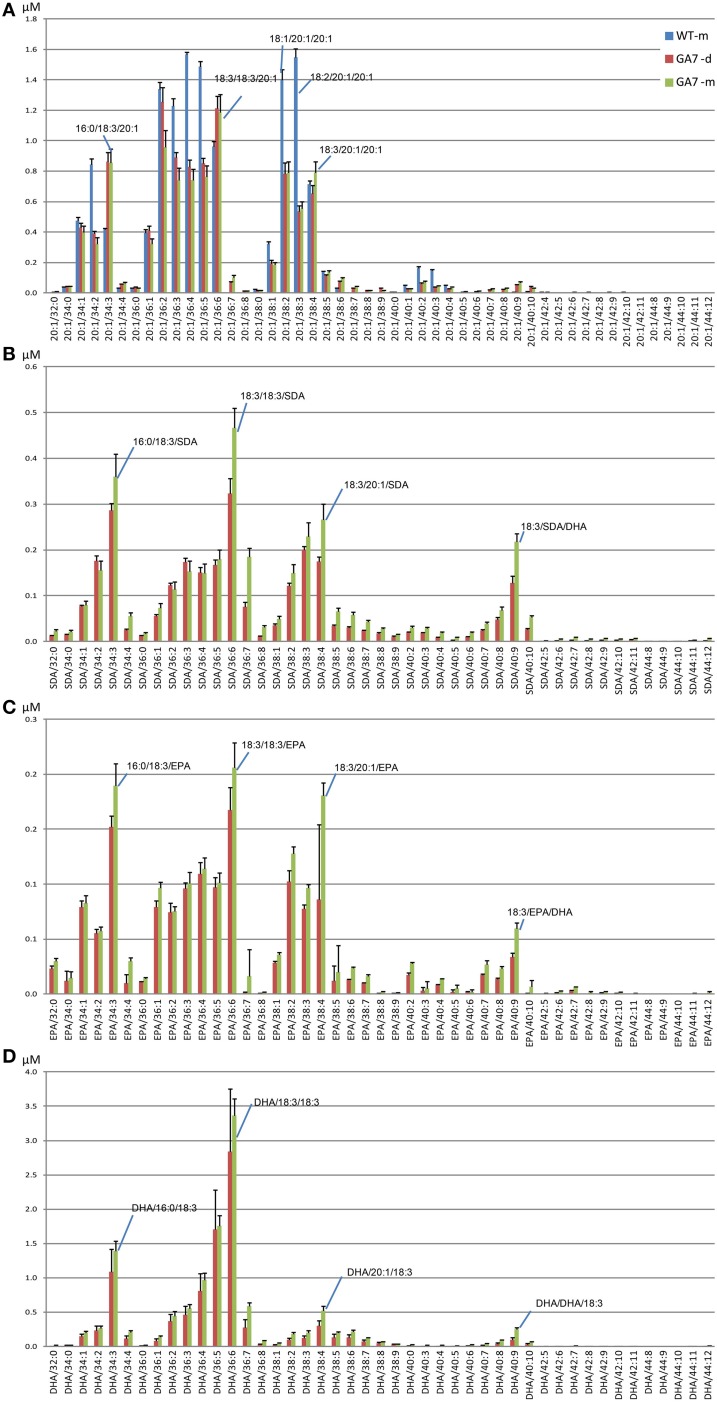
**Abundance of different triacylglycerol (TAG) species in the control (WT) and the transgenic (GA7) *Arabidopsis* seeds**. TAG species are labeled in the format of TAG containing target/X:Y, where target will be 20:1 **(A)**, SDA **(B)**, EPA **(C)** or DHA **(D)**, with the other two acyl chains containing X number of total carbons and Y number of total double bonds. Data are shown as the mean of triplicate analysis with the error bars representing the standard deviations. Predominant TAG species within the subgroups are indicated.

DHA-containing TAG species were only found in the GA7 developing or mature seeds. The accumulation of LC-PUFA-containing TAG was at the expense of 20:1-containing TAG (Figure 7). This is in line with previous results reported for total lipids from the GA7 seeds showing that 20:1 was significantly reduced (Petrie et al., 2012). In GA7, most of the 20:1-containing TAG species were reduced when compared to WT, except for the TAG species that also contained ALA or LC-PUFA. For example, TAG 20:1/34:3 (TAG 20:1/16:0/18:3) and TAG 20:1/36:6 (TAG 20:1/18:3/18:3) were increased in GA7 compared to WT (Figure 8A). SDA-, EPA- and DHA-containing TAGs were only found in the GA7 developing or mature seeds. As expected, TAG species 20:1/40:5 to 20:1/40:10, consisting of 20:1 plus LC-PUFA beyond 20:3 in the pathway, were absent in WT. This is in contrast to their accumulation, although at low levels in GA7. The highest 20:1-containing TAG species in GA7 were TAG 20:1/36:2 and 20:1/36:6.

TAG species in the GA7 developing and mature seeds containing SDA, EPA or DHA that were absent from WT, are shown in Figures 8B–D. The levels of TAG SDA/34:3, TAG SDA/36:6, TAG SDA/36:7, TAG SDA/38:4 and TAG SDA/40:9 increased significantly from the developing seeds to the mature seeds. There was no significant change in profile of EPA- or DHA-containing TAG species in the developing or mature seeds. EPA accounted for only 1.8%, compared to 15.1% of DHA in total seed lipids.

### Major novel ω3 LC-PUFA containing TAG species in the transgenic seeds

The most abundant TAG species among the four analyzed groups in mature GA7 seeds were the TAG species that also contained 18:3, i.e., TAG 20:1/36:6, TAG SDA/36:6, TAG EPA/36:6, TAG DHA/36:6. This was followed by TAG 20:1/34:3 and TAG 20:1/36:2, TAG SDA/34:3 and TAG SDA/38:4, TAG EPA/34:3 and TAG EPA/38:4, or TAG DHA/34:3 and TAG DHA/36:5. Very low amounts of tri-DHA TAG was also detected in the GA7 mature seeds. This highlights the utility of LC-MS based lipid profiling as this finding would be impossible with GC-MS based protocols. These identities were confirmed by MS/MS analysis (Figure S4) and summarized in Table 2.The results also suggested a likely preference of LC-PUFA combined with 18:3 in TAG. Based on the TAG fatty acid profile in the mature seed of GA7 which was dominated by 18:3, 22:6, 16:0, and 20:1 (Petrie et al., 2012), the TAG species distribution among all the DHA containing TAG species was calculated, if they were randomly associated. The TAG species that were significantly higher in measured association than the randomized prediction mainly contained 18:3 (Table S2). In contrast, the DHA containing TAG species combined with other dominant fatty acids like 16:0 and 20:1 were significantly lower than the randomized prediction. The predicted tri-DHA in all the DHA containing TAG species could be 0.9%, but only 0.1% was measured. These suggested that DHA distribution in TAG was not randomly. Non-random pattern of elongated acyl chains that are incorporated into TAG in Arabidopsis seed is recently discovered by LC-MS (Li et al., 2014).

**Table 2:** **MS/MS fragmentation and structure analysis of the selected TAG species**.

**TAG species**	**Precursor**	**Neutral loss**	**Major or only composition of TAG**
TAG 20:1/34:3	900.77	16:0, 18:3, 20:1	16:0/18:3/20:1
TAG 20:1/36:6	922.75	18:3, 20:1	18:3/18:3/20:1
TAG 20:1/38:4	954.81	18:3, 20:1	18:3/20:1/20:1
TAG SDA/34:3	866.72	16:0, 18:3, SDA	16:0/18:3/SDA
TAG SDA/36:6	888.70	18:3, SDA	18:3/18:3/SDA
TAG SDA/38:4	920.77	18:3, SDA, 20:1	18:3/SDA/20:1
TAG SDA/40:9	938.72	18:3, SDA, DHA	18:3/SDA/DHA
TAG EPA/34:3	892.74	16:0, 18:3, EPA	16:0/18:3/EPA
TAG EPA/36:6	914.72	18:3, EPA	18:3/18:3/EPA
TAG EPA/38:4	946.78	18:3, 20:1, EPA	18:3/20:1/EPA
TAG EPA/40:9	964.74	18:3, EPA, DHA	18:3/EPA/DHA
TAG DHA/36:6	940.71	18:3, DHA	18:3/18:3/DHA
TAG DHA/34:3	918.72	16:0, 18:3, DHA	16:0/18:3/DHA
TAG DHA/38:4	972.77	18:3, 18:4, 20:0, 20:1, DHA	18:3/20:1/DHA, 18:4/20:0/DHA
TAG DHA/40:9	990.72	18:3, DHA, DHA	18:3/DHA/DHA
TAG DHA/44:12	1040.60	DHA	DHA/DHA/DHA

The positional distribution of the three fatty acids in the abundant TAG species could not be resolved by LC-MS analysis. Other studies using NMR analysis of DHA in *Arabidopsis* seed oil have shown that DHA is preferentially positioned at *sn*-1/3 (Petrie et al., 2012). The most abundant DHA-containing DAG or TAG were DAG DHA/18:3 or TAG DHA/18:3/18:3, implying the 2nd 18:3 fatty acid might be at the *sn*-3 position, thus the DHA might be at *sn*-1 position. It should be noted that 18:3ω6 (GLA) and 18:3ω3 (ALA) could not be distinguished by LC-MS analysis, GA7 had very low levels of GLA in total seed lipids compared to the high levels of ALA. Therefore, the abundant C18:3 in the DHA-containing TAG might be ALA. The observed low amount of di-DHA PC, di-DHA DAG and tri-DHA TAG also suggested that the transgenic GA7 was able to incorporated DHA onto all three positions of TAG albeit at low levels. A similar observations showing low amounts of tri-EPA TAG (1.5% of total EPA-containing TAG) was also reported from the transgenic *Camelina sativa* seed expressing the EPA synthesis pathway (Ruiz-Lopez et al., 2013a). DHA esterified onto *sn*-1 position would most likely be contributed by GPAT enzyme, which catalyzes the acylation of glycerol-3-phosphate with acyl-CoA. Although the acyl-CoA pool was not characterized in this study, incorporation of 15% of DHA in GA7 TAG together with the fact that tri-DHA TAG was also detected, suggested that the appearance of LC-PUFA in acyl-CoA pool did occur. Furthermore, the final step of acylation from DAG to TAG can occur by a number of distinct TAG assembly pathways, including acyl-CoA dependent DGAT, or by the transferring of the acyl group from *sn*-2 of PC to *sn*-3 of DAG by phospholipid:diacylglycerol acyltransferase (PDAT) (Dahlqvist et al., 2000). LC-MS analysis described here was not able to indicate which one of these routes was the major contributor to the TAG assembly. However, the endogenous *Arabidopsis* AtDAGT2 was shown to prefer to PUFA for the DAG to TAG conversion (Zhou et al., 2013). The AtDGAT2 might favor the assembly of more 18:3 onto DHA containing-DAG, resulting in the high proportion of TAG DHA/18:3/18:3 observed in our study.

### DHA from synthesis to storage

In the mature seeds of GA7, 11.7% DHA was accumulated in total PC (Table 1). In the total seed lipids of GA7, DHA levels increased to 15.1%. GA7 had the substantial increase of PC 34:3 (PC 16:0/18:3) and PC 36:6 (PC 18:3/18:3), along with significant amounts of TAG DHA/18:3/18:3 and TAG DHA/16:0/18:3. These observations suggest efficient flux from PC 16:0/18:3 and PC 18:3/18:3 to the TAG pool. Indeed, analysis of 18:3-containing DAG species showed DAG 18:3/16:0 and DAG 18:3/18:3 had a significant increase in GA7 when compared to WT (data not shown). This observation was in agreement with that previously proposed for the PC-derived DAG contributing to synthesis of PUFA-containing TAG (Bates and Browse, 2012).

Recently, Ruiz-Lopez et al. (2013a) reported a maximum transgenic production of 14% DHA in *C. sativa*. By transgenically expressing a different set of seven enzymes, they found the DHA accumulation in PC was higher than DAG, with even lower levels in TAG, indicating the existence of an inefficient flux of DHA into TAG. This might reflect the different set of enzymes used in these two studies, or due to the different host background. In summary, the accumulation of high levels of DHA in transgenic *Arabidopsis* seed oil was accompanied with enhanced levels of PL especially PC, DHA-containing DAG and TAG species, as well as the decreased levels of 20:1-containing DAG and TAG. The work also showed evidence that there is a preference of engineered LC-PUFA, especially DHA, in neutral and phospholipid containing 18:3. This study demonstrates that LC-MS analysis of lipidome is a invaluable tool for gaining insights into how the engineered fatty acid combine with other fatty acids in lipids of transgenic seeds.

## Author contributions

Conceived and designed the experiment: Xue-Rong Zhou, Surinder P. Singh. Performed the experiment: Xue-Rong Zhou, Damien L. Callahan, Pushkar Shrestha, Qing Liu, James R. Petrie. Analyzed the data: Xue-Rong Zhou, Damien L. Callahan, Pushkar Shrestha, Surinder P. Singh. Contributed reagents/materials/analysis tools: Damien L. Callahan, Pushkar Shrestha. Wrote the paper: Xue-Rong Zhou, Surinder P. Singh. All authors revised the draft and approved the final manuscript.

### Conflict of interest statement

The authors declare that the research was conducted in the absence of any commercial or financial relationships that could be construed as a potential conflict of interest.

## Supplementary material

Figure S1 **MS/MS analysis of phosphotidylcholine (PC) precursor at m/z 878.6 with a PC head group ion of m/z 184.2**. Y axis represents the response of ion scan. The low abundant fragment at *m/z* 568.3 is due to the neutral loss of 22:6, thus confirming the identity as PC 22:6/22:6.

Figure S2 **Low abundance phosphatidylserine (PS) pool in the developing and the mature *Arabidopsis* seeds**. The PS are annotated similarly to PC. Data are shown as the mean of triplicate analysis with the error bars representing the standard deviations.

Figure S3 **The abundances of long-chain polyunsaturated fatty acids (LC-PUFA) containing diacylglecerol (DAG) in the WT and GA7 *Arabidopsis* seeds**. Data are shown as the mean of triplicate analysis with the error bars representing the standard deviations.

Figure S4 **Product ion spectra for identification of selected lipid species. (A)** Fragmentation of the [M+NH_4_^+^]^+^ ion at 940.75 *m/z* showing the neutral losses of 18:3 and 22:6, represents a triacylglycerol (TAG) composed of 18:3/18:3/DHA. **(B)** Fragmentation of the [M+NH_4_]^+^ ion at 922.79 *m/z* showing the neutral losses of 18:3 and 20:1, represents a TAG composed of 18:3/18:3/20:1. **(C)** Fragmentation of the [M+NH_4_]^+^ ion 1040.59 *m/z* showing the neutral loss of 22:6, represents a TAG composed with tri-DHA.
