# Supplementary figures and images for: Lipidomic analysis of Arabidopsis seed genetically engineered to contain DHA
Source: Front Plant Sci. 2014 Sep 1;5:419. doi: 10.3389/fpls.2014.00419 (PMC4150447; doi:10.3389/fpls.2014.00419)

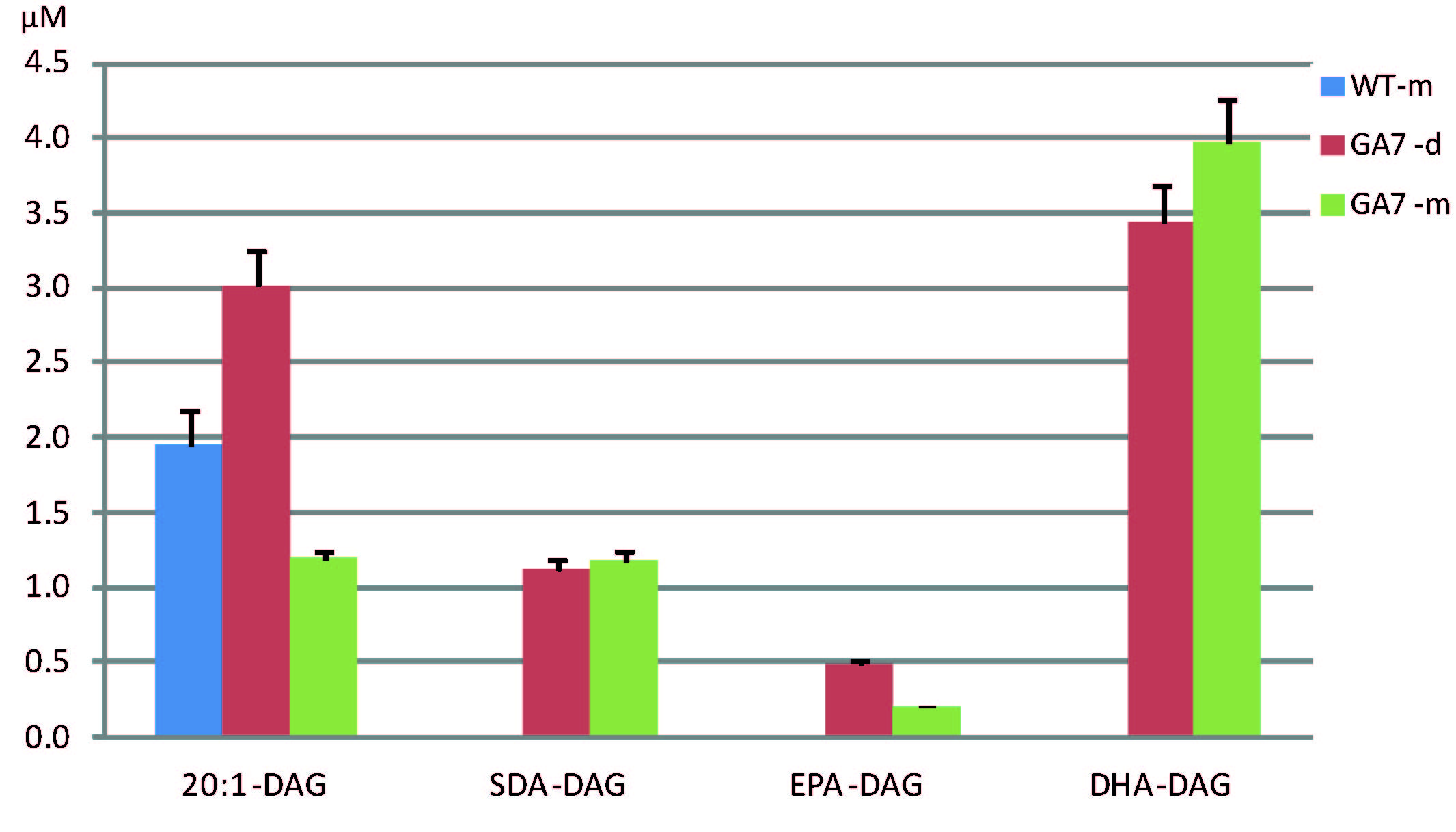

Supplement: Figure S1 — MS/MS analysis of phosphotidylcholine (PC) precursor at m/z 878.6 with a PC head group ion of m/z 184.2. Y axis represents the response of ion scan. The low abundant fragment at m/z 568.3 is due to the neutral loss of 22:6, thus confirming the identity as PC 22:6/22:6. [file Presentation1.ZIP › Figure S3.JPEG]

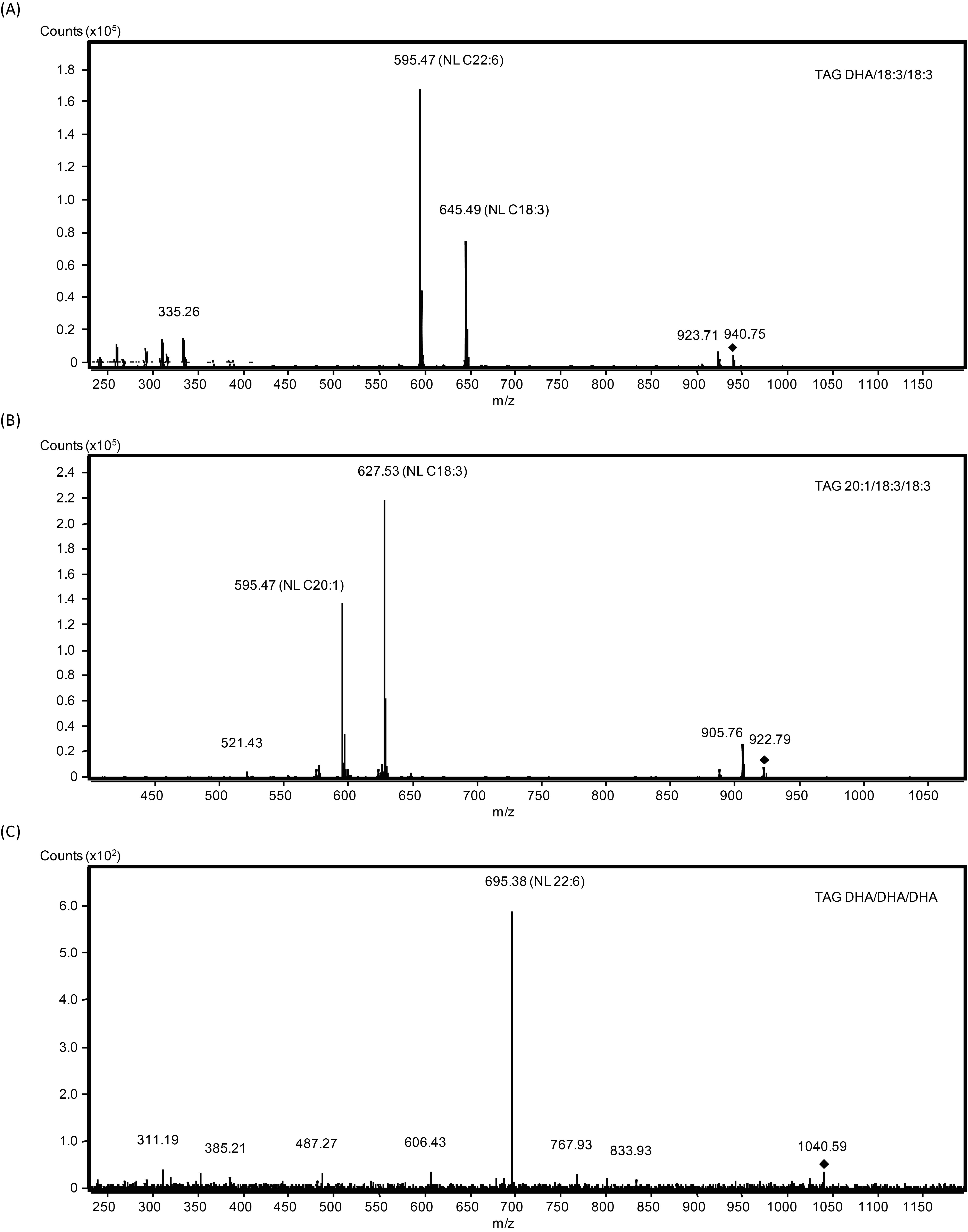

Supplement: Figure S1 — MS/MS analysis of phosphotidylcholine (PC) precursor at m/z 878.6 with a PC head group ion of m/z 184.2. Y axis represents the response of ion scan. The low abundant fragment at m/z 568.3 is due to the neutral loss of 22:6, thus confirming the identity as PC 22:6/22:6. [file Presentation1.ZIP › Figure S4.JPEG]

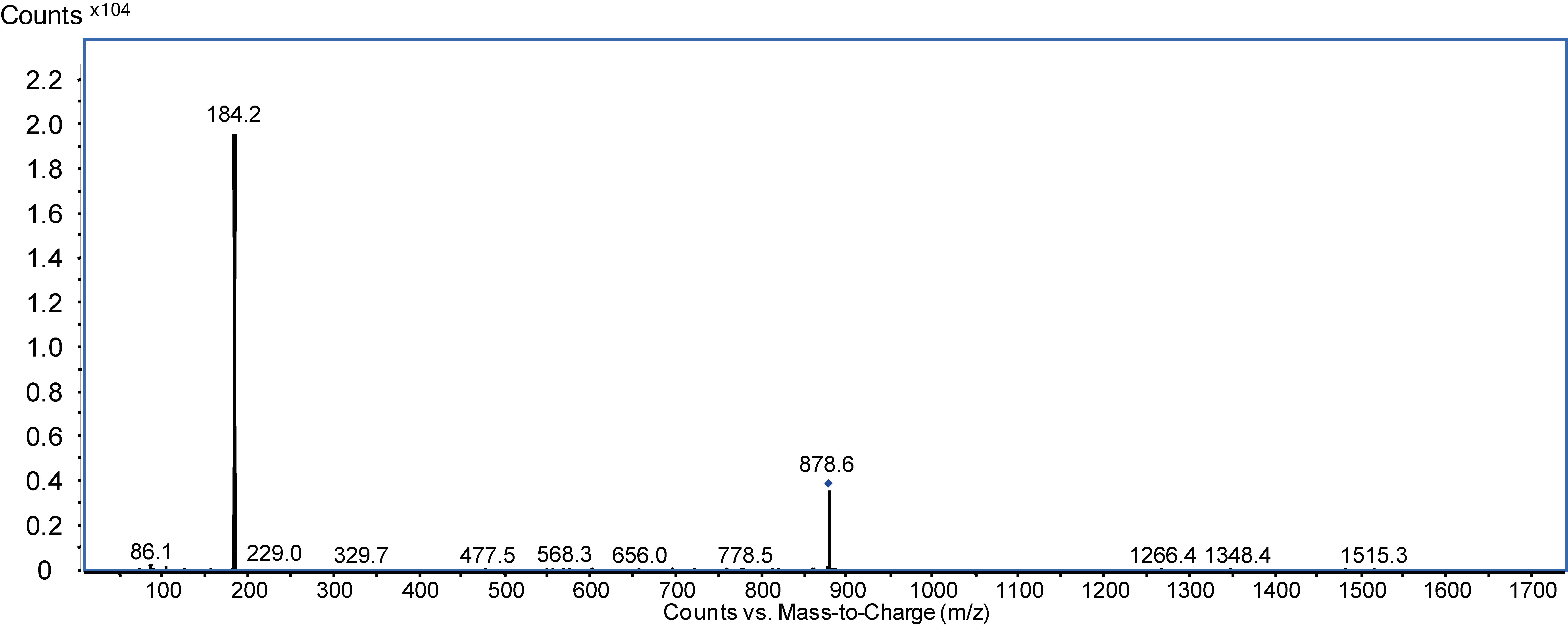

Supplement: Figure S1 — MS/MS analysis of phosphotidylcholine (PC) precursor at m/z 878.6 with a PC head group ion of m/z 184.2. Y axis represents the response of ion scan. The low abundant fragment at m/z 568.3 is due to the neutral loss of 22:6, thus confirming the identity as PC 22:6/22:6. [file Presentation1.ZIP › Figure S1.JPEG]

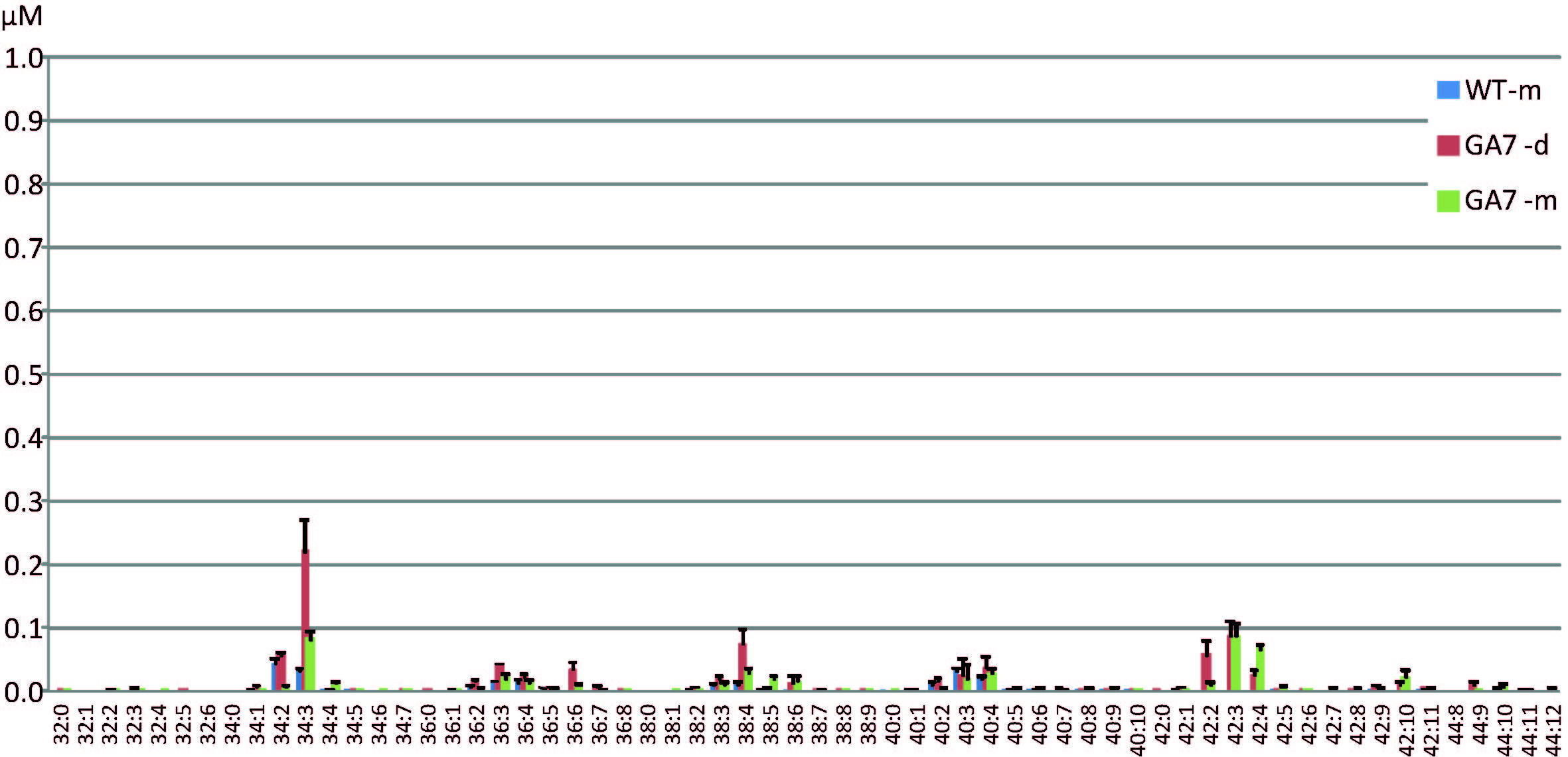

Supplement: Figure S1 — MS/MS analysis of phosphotidylcholine (PC) precursor at m/z 878.6 with a PC head group ion of m/z 184.2. Y axis represents the response of ion scan. The low abundant fragment at m/z 568.3 is due to the neutral loss of 22:6, thus confirming the identity as PC 22:6/22:6. [file Presentation1.ZIP › Figure S2.JPEG]
